# Microstructure and Properties of Inconel 625 Fabricated Using Two Types of Laser Metal Deposition Methods

**DOI:** 10.3390/ma13215050

**Published:** 2020-11-09

**Authors:** Jan Dutkiewicz, Łukasz Rogal, Damian Kalita, Katarzyna Berent, Bogdan Antoszewski, Hubert Danielewski, Marek St. Węglowski, Magdalena Łazińska, Tomasz Durejko, Tomasz Czujko

**Affiliations:** 1 Institute of Metallurgy and Materials Science, Polish Academy of Sciences, PAS, 25, Reymonta St., 30-059 Krakow, Poland; rogal.l@imim.pl (Ł.R.); kalita.d@imim.pl (D.K.); 2 Academic Centre for Materials and Nanotechnology, AGH University of Science and Technology, 30, Mickiewicza Av., 30-059 Kraków, Poland; berent@agh.edu.pl; 3 Laser Research Center, Department of Mechatronics and Mechanical Engineering, Kielce University of Technology, 25-314 Kielce, Poland; ktrba@tu.kielce.pl (B.A.); hdanielewski@tu.kielce.pl (H.D.); 4 Lukasiewicz Research Network, 16-18 Bl. Czesława Str., 44-100 Gliwice, Poland; Marek.Weglowski@is.gliwice.pl; 5 Department of Materials Technology, Military University of Technology, 2 Gen. Kaliskiego Str., 00-908 Warsaw, Poland; magdalena.lazinska@wat.edu.pl (M.Ł.); tomasz.durejko@wat.edu.pl (T.D.)

**Keywords:** Inconel 625, laser engineered net shaping (LENS), CO_2_ laser deposition

## Abstract

The effect of using two different deposition systems on the microstructure and mechanical properties was studied in this paper. For this purpose, laser-engineered net shaping (LENS) and high-power CO_2_ laser deposition processes were applied to fabricate Inconel 625 samples. The microstructure of the Inconel 625 produced by both additive techniques was characterized using light microscopy (LM), scanning electron microscopy (SEM), electron backscatter diffraction (EBSD), and transmission electron microscopy (TEM). The mechanical properties were characterized by tensile tests and microhardness measurements. High-power laser application resulted in a strong <100> build texture, while, at low powers, the {011} <100> Goss component increased. Both types of deposited materials showed dendritic microstructures with Ti-, Mo-, and Nb-rich zones at the cell boundaries, where numerous precipitates (Nb_2_C, NbC, titanium carbides, Nb_3_Ni, and NbNiCr) were also observed. It was also noted that both variants were characterized by the same slope with a proportional length, but the Inconel 625 fabricated via LENS showed a higher average yield strength (YS; 524 MPa vs. 472 MPa) and ultimate tensile strength (UTS; 944 MPa vs. 868 MPa) and lower elongation (35% vs. 42%) than samples obtained with the high-power CO_2_ laser deposition process.

## 1. Introduction

Inconel 625 is a superalloy that is often applied in the aircraft engine industry due to its excellent corrosion resistance and very good high-temperature strength properties. In most cases, parts are manufactured using plastic working; however, due to a high strain hardening ability, plastic working is difficult [1]. Therefore, several recent attempts have been reported to fabricate components from Inconel 625 alloys using additive manufacturing methods. In the majority of papers, selective laser melting (SLM) deposition was employed [2,3,4,5,6], and, only in a few works, other methods, such as pulsed plasma arc deposition [7,8], wire + arc additive manufacturing (WAAM) [9,10,11], and electron beam melting (EBM) [12], were applied. In most cases, Inconel 625, additively manufactured by the SLM or EBM techniques, shows a dendritic columnar microstructure with elongated grains in the build direction [2,4,5,13,14] and a texture characterized by a strong Goss {011} <100> and weak cube {001} <100> components [14]. However, researchers describe the structure formation as <100> epitaxial growth [4]. Moreover, Le et al. [2] suggested that 80% of the grains were oriented parallel to the (111) and (101) planes within a certain variation of the orientation. Similar observations were also reported for other types of Inconel alloys, such as 718 fabricated using the SLM method, where fast growth for some grains with the <100> orientation parallel to the heat flow direction was suggested and highly inhomogeneous and strongly textured microstructures with long grains elongated in the (001) direction were observed [15].

In addition to the microstructure and texture, many researchers have studied the morphology and crystallographic structure of precipitates in the Inconel 625 alloy after additive manufacturing [2,3,4,5,7,9,10,13,16,17,18,19,20]. This problem was also investigated using a theoretical simulation of precipitation for different degrees of segregation and at different temperatures, which enabled construction of a time–temperature–transformation diagram (TTT) for additively manufactured Inconel 625. It was concluded that there was a large amount of segregation, i.e., the Nb- and Mo-rich interdendritic regions, and fast precipitation of δ Ni_3_Nb and MC phases [21]. As confirmed in [7], the primary liquid → γ reaction causes the accumulation of Nb, Mo, C, and Ti elements in interdendritic regions and grain boundaries. Thus, the Laves phase, MC carbides (including Cr_23_C_6_, NbC, and TiC), and δ-Ni_3_Nb can precipitate in these regions, affecting the mechanical properties [2,7]. However, depending on the cooling rate in a laser powder bed fusion additively deposited sample [5], no identifiable peaks other than those of the face-centered cubic (FCC) phase were observed, confirming that the microstructure almost entirely comprised a single phase. Moreover, most papers on AM of Inconel 625 after deposition showed the presence of the Ni_3_Nb phase [2,16,17]. The size of the MC carbides and the volume fraction of the δ phase increased with increasing post-deposition heat treatment time [4,5,6,10]. After annealing above 800 ℃, M_23_C_6_ carbide and Laves phases were also observed [7,17]. In the case of laser metal deposition (LMD), a mean hardness value of 220 HB was obtained, similar to that observed in material fabricated by SLM [4], which is comparable with that for commercially available as-rolled Inconel 625, which is 175–240 HB.

There are also discrepancies in the literature concerning the porosity of samples after additive manufacturing [2,6,22]. The formation of balling [2,23] is mainly caused by the high viscosity and surface tension of a molten material, which tends to minimize its surface area by forming liquid spheres. Balling may also contribute to the pores in fabricated parts [23]. Clusters of unmelted powders, which may be trapped in the cavities caused by an unevenly distributed layer thickness, significantly decrease the wettability of the melt pool to the previous layer, also causing pores. In [7], it was reported that, for laser metal deposition of Inconel 625, the maximum hardness values near 290 HV corresponded to a residual porosity of 0.05%, while, in the case of SLM, maximum hardness values of 215 HV were associated with a residual porosity of 0.15%. Porosity studies presented in [22] showed a slight increase in the porosity in SLM-deposited Inconel 625 as the powder flow increased. In addition, a low carrier gas flow rate caused the lowest porosity therein. The hardness for parts fabricated with SLM was very high (254 HV) due to the very fine microstructure and high degree of supersaturation of strengthened elements [4,6].

Substantial stress relief and grain coarsening were observed after solution heat treatment, particularly at 1250 °C, because of the formation of subgrains, precipitates, and dislocation networks. However, the 1100/1250 °C heat treatment did not dissolve microsegregated precipitates and carbides completely. In [10], it was reported that the strength and hardness increased after heat treatment of SLM materials, and the values were comparable with those of wrought Inconel 718 alloy. Their ductility decreased significantly compared with that of the as-fabricated material due to precipitation of fine γ′ and γ″ strengthening phases and needle-like δ phases. Moreover, in Inconel 740 after an aging treatment at 800 °C for 16 h and air cooling, fine γ′ precipitates and small carbide particles were identified at the grain boundaries. The hardness increased up to 304 HB, and the impact toughness decreased to 15 J after aging. Inconel 625 showed significant hardening during deformation at room temperature and hardening during aging at a temperature of only 550 °C [16], while recovery also started producing a significant effect at this temperature [1]. The effect of carbide and intermetallic precipitates formed during AM using both methods from Inconel 625 was studied using scanning and transmission electron microscopy to determine their crystal structure, type of interface, and density, as well as composition of solid solution, on mechanical properties, as suggested in [24].

Recent studies of Inconel alloys additive manufacturing showed inconsistent results concerning the microstructure, texture, and mechanical properties. The differences in the fusion mechanism in laser deposition methods affect those aspects and, therefore, properties of manufactured materials [25,26,27,28]. Some publications presenting a comparison of discussed Inconel 625 prototyping methods referred to differences in microstructure [6,29]. However, there is a lack of studies related to a comprehensive metallographic analysis, combined with mechanical properties where a comparison of material properties is related to process parameters. The aim of the work was to perform a comparative study of an Inconel 625 alloy fabricated using two competitive laser metal deposition (LMD) methods: (i) a high-power CO_2_ laser with a TruLaserCell 1005 machine using powder injection from the nozzles into the melt pool protected by helium gas flow (DLD), and (ii) a laser-engineered net shaping (LENS) MR-7 system (Optomec, Albuquerque, NM, USA) equipped with a 500 W fiber laser and a similar powder flow, where the process was conducted in the chamber under a purified argon atmosphere. Advanced microstructural analysis specified which technique gave better properties for specific applications, and which type of material defects can occur for those fabrication methods.

## 2. Materials and Methods

**Figure 1:**
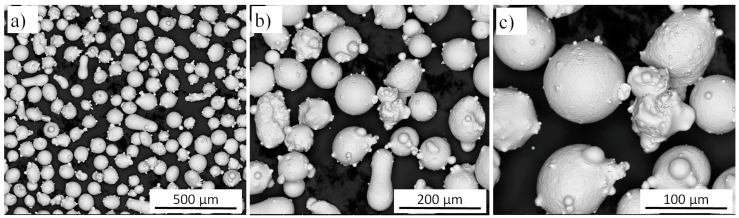
The morphology of Inconel 625 powder particles shown at different magnification: (**a**) 200×; (**b**) 500×; (**c**) 1000×.

In the present study, two different laser metal deposition systems were used to fabricate components comprising an Inconel 625 alloy. A gas atomized powder (delivered by LPW Technology, Runcorn, UK) with particle sizes in the range of 45–106 μm (Figure 1a) and chemical composition presented in Table 1 was used. The shape of Inconel 625 powder particles was mostly spherical with microsatellites attached to them (Figure 1b). However, several elongated (Figure 1b) and with irregularly shaped (Figure 1c) particles were also observed. 

In the first method, the deposition process was performed using a high-power CO_2_ laser with a Trumpf Lasercell 1005 machine (Trumpf GmbH, Ditzingen, Germany) with a laser cladding head that had a focal length of 270 mm and was integrated with a coaxial powder feeder type GTV PF 2/2. The metallic powder was transported through three nozzles by helium gas to the laser beam focal point. Helium gas was applied as a carrier gas instead of the commonly used Ar because of its high thermal conductivity. Multiple layers were deposited on an Inconel 625 sheet plate. The following parameters were used to develop the cuboid sample: a laser power of 2.275 kW, laser spot diameter of 2.0 mm, scanning speed of 800 mm/min, and powder feed of 15 g/min. Subsequent layers of the alloy were formed in the shape of cuboids with dimensions of 16 mm × 16 mm × 45 mm. Layers were deposited alternately until the assumed geometry was attained. The working head was oriented perpendicular to the substrate material surface. Since Inconel 625 has a very low thermal conduction coefficient and the thermal exchange is relatively slow, no additional preheat or postprocess heat treatment was performed. The prototype solid material was machined to form cylindrical samples for further characterization. The second type of deposition process was performed using a laser-engineered net shaping (LENS) LENS MR-7 system (Optomec, Albuquerque, NM, USA) equipped with a 500 W fiber laser. To obtain cylindrical samples with a diameter of 20 mm and height of 10 mm, the following parameters were applied: a laser power of 400 W, laser spot diameter of 1.2 mm, working table feed rate of 600 mm/min, powder flow rate of 15 g/min, and single layer thickness of 0.3 mm. Moreover, the process was performed in the chamber under a purified argon atmosphere (O_2_ and H_2_O < 10 ppm). Additionally, for both laser methods, the temperature profile of the melt pool for a middle layer (at the half-height of the samples) was recorded, and then the cooling rate was calculated. The exposure time (a measurement time) was 15 ms with a 5 s interval time (an average of five temperature profiles for each sample were acquired). The deposition parameters for both methods were adopted on the basis of a preliminary investigation, where the main criteria were a reduction in thermal energy supplied into material and preservation of a high shape coefficient (height relative to width of single bead layer) with no defects (cracking, porosity or spatters) detected.

Optical observations of the fabricated samples were performed using a Leica DMIRM microscope (Leica, Wetzlar, Germany) on the previously etched samples using Kroll’s reagent. Detailed microstructural characterization was carried out with electron backscatter diffraction (EBSD) measurements on a Versa 3D FEG scanning electron microscope (FEI, Hillsboro, OR, USA) equipped with an EDAX Hikari CCD camera. Chemical characterization of the samples was performed using an EDAX Apollo XP energy-dispersive X-ray spectrometer (EDAX, Berwyn, PA, USA). Additionally, the microstructure analysis of the investigated materials was performed using a Tecnai FEG G2 F20 Super Twin transmission electron microscope (FEI, Hillsboro, OR, USA). The thin foils for transmission electron microscopy (TEM) observations were prepared by electropolishing discs with a thickness of 100 μm in an electrolyte containing 10 mol.% HClO_4_ in methanol (with a voltage of 20 V and temperature of −20 °C).

The tensile tests were performed on an Instron 8501 testing machine with an initial strain rate of 10^−3^ s^−1^. The load force–elongation response of the material was recorded using an air-cooled extensometer. All samples for the tensile tests with a gauge length of 20 mm and diameter of 5 mm were machined from the Inconel 625 samples that were fabricated with the high-energy CO_2_ laser and LENS methods and then machined using an ST-20 CNC HAAS lathe. For each technological variant, three samples were tested. 

Additionally, to validate the effect of the cooling rate on the mechanical properties, Vickers microhardness measurements using a Shimadzu HMV-G micro Vickers hardness tester were conducted with 100 g of load and 10 s dwell time for each single indentation. The microhardness was tested at least 10 times at different locations on the sample cross-sections.

## 3. Results and Discussion

Samples made with two different additive manufacturing methods, namely, the high-energy CO_2_ laser and LENS systems, were subjected to structural and mechanical properties tests.

### 3.1. Substrate Material Characterization

**Figure 2:**
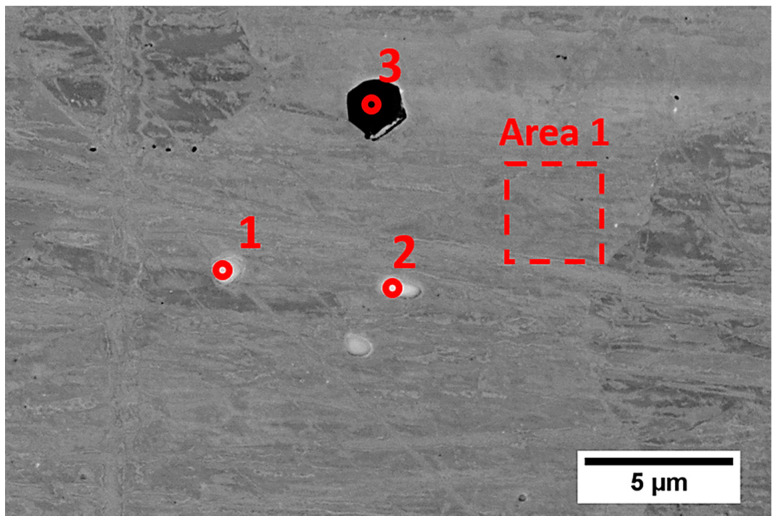
SEM (BSE) micrograph of precipitations observed in the substrate material.

The structural characterization of the substrate material together with chemical composition analysis in microscopic areas (Figure 2 and Table 2) showed the presence of visible and relatively large particles of other phases (bright and dark precipitates) that were enriched in Nb and Ti. On the basis of the chemical compositions and literature studies, we can presume that they comprised TiC or (Nb,Ti)C, which were also observed in [7] and [19], respectively.

One can see that bright particles were rich in Nb, Mo, Ti, and C, which suggests the presence of carbides, as observed in IN625 AM alloys [2,4,7], while dark particles (marked 3) that were rich in Ti and Nb without carbon may be one of the Laves phases or other intermetallic phases also observed in Inconel 625 [4,5].

### 3.2. Microstructure of Inconel 625 Deposited by CO_2_ Laser 

**Figure 3:**
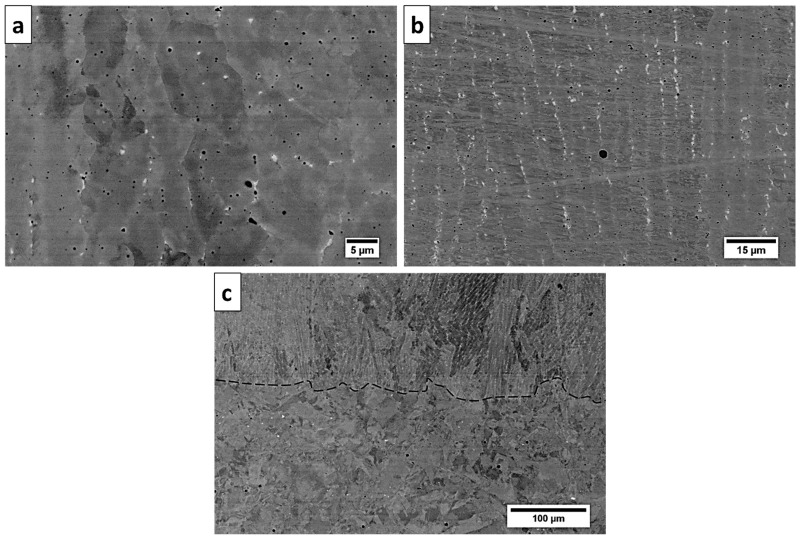
SEM micrograph of Inconel 625 deposited using a high-power CO_2_ laser at three different magnifications: (**a**) low-magnification observations show the cross-section of a substrate and deposited material; (**b**) higher magnification of deposited material; (**c**) highest magnification showing the precipitations and porosity in the microstructure.

The microstructure of the Inconel 625 deposited using a high-power CO_2_ laser is presented in Figure 3a–c, which show SEM micrographs taken near the interface between the substrate and deposited material. One can see the interface between the substrate and the deposited material, where the substrate comprised regular uniaxial grains with a size near 50 μm, and the deposited material consisted of large columnar grains that grew perpendicular to the surface. Within the substrate and deposited material, many pores formed due to thermal shock and decreased the mechanical properties. The formation of pores was reported earlier in [6,22] in samples fabricated by directed energy deposition (DED) and SLM, where the density of pores was related to the powder feeding rate and power of the laser, respectively. In our case, pores also formed near the interface within the substrate.

**Figure 4:**
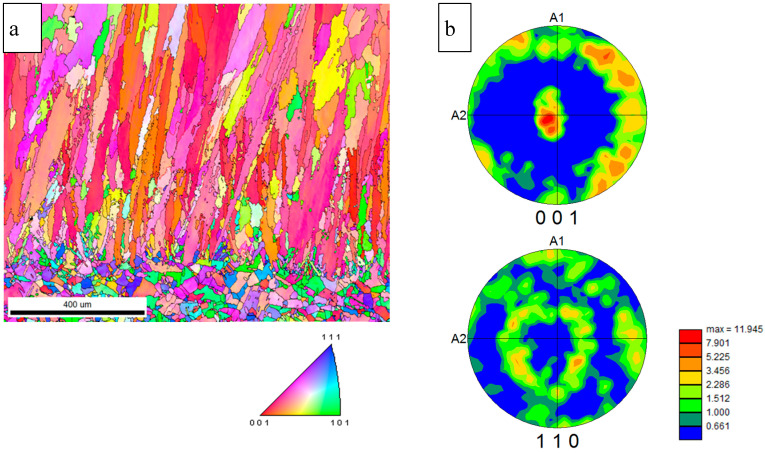
The electron backscatter diffraction (EBSD) results for samples fabricated using high-power CO_2_ laser: (**a**) inverse pole figure (IPF) maps for the substrate and the deposited material and (**b**) 001 and 111 pole figures rotated to represent the deposition surface; the color scale bar represents the intensity of the texture.

The results of electron backscattered diffraction (EBSD) analysis made on the cross-section of the substrate and material deposited using a high-power CO_2_ laser are presented in Figure 4. One can see that the grains had a well-developed axial [001] <110> texture indicated by the orange and red colors in the center compared to various colors of a random orientation (blue) (Figure 4b). A similar type of texture with long axes for most of the grains aligned in the <001> direction and a cube texture was observed by C. Li et al. and S. Li et al. in Inconel 625 [2,4] as well as by Popovich et al. and Wang and Chou in Inconel 718 [15,25], where the <100> orientation was parallel to the heat flow direction and the intensity of the <100> texture increased with the laser power [4]. This effect may also be strengthened by the high energy density of the CO_2_ laser and melting conditions promoted to form a deep melt pool, similar to that observed by Wang and Chou [30] and Robichaud et al. [31]. Moreover, from the calculated grain boundary misorientation angles, the grain boundaries in the deposited layer were preferentially high-angle grain boundaries (HAGBs) with a misorientation from 25–50°, while those of the substrate were mostly either low-angle grain boundaries (LAGBs) or misoriented near 60°. According to the distribution statistics of the grain misorientation, the grain boundary ranges of the LAGBs (28.1%) and HAGBs (71.9%) in the deposited material were observed. Most of the grains were separated by high-angle grain boundaries, which was probably due to the high energy of the CO_2_ laser beam. The distribution of high-angle boundaries is similar to that reported in [25], where authors suggested that the presence of HAGBs could strongly affect both the density and the distribution of cracks in an as-fabricated material and that the as-deposited material is very susceptible to ductile tip cracking at HAGBs. The statistical results for the substrate show that most misoriented grain boundaries were composed of HAGBs (76.1%) and LAGBs (23.9%). LAGBs are generally described as an array of dislocations and, thus, are representative of an accumulation of lattice defects in a material. The calculated average size of elongated crystallites in the deposited material was 85 μm and that of the substrate grains was 27 μm, which is similar to the result observed for material fabricated using EBAM [11] and SLM [2] methods. However, Marchese et al. reported that Inconel 625 fabricated using the SLM technique [6] had much smaller grains near 1 μm. One can see the extension of the length of the grains increasing with distance from the interface, and some grains reached even 1000 μm in size, which is still less than the 3 mm length of grains reported for Inconel 718 builds obtained using the direct laser additive manufacturing process [32] and phases observed in Inconel 625 [11,16]. The equiaxial grains at the bottom of the micrograph belong to the substrate from the annealed sheet of Inconel 625. Their shape and size did not change much during deposition, only some of them started to grow in the perpendicular direction to the surface, but finally most of them showed texture close to axial <001>.

**Figure 5:**
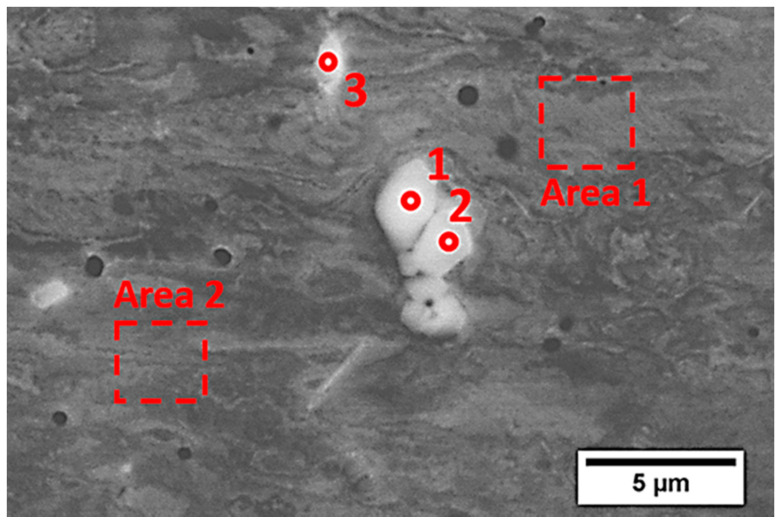
SEM (BSE) micrograph of precipitations observed in the material deposited using high-power CO_2_ laser.

A scanning electron microscopy (SEM) micrograph in BSE mode of the material deposited using a high-power CO_2_ laser is presented in Figure 5. Similar to other studies [2,4,5,7], fine submicron particles were frequently observed in the deposited material. Considering the size of the observed precipitates, their quantitative chemical analysis might be complicated due to the XRD signal originating from the matrix material. The chemical composition of the fabricated material (Table 2) was similar to that of the delivered powder material, except for elements such as iron and aluminum, which had a lower content. Particles marked in Figure 5 as 1 and 2 showed more Nb, Mo, Ti, and C (Table 3), which most likely indicated the presence of MC carbides, as previously observed in an additively manufactured Inconel 625 alloy [2,4,7]. The precipitate marked as point 3 had an increased amount of Ni, Nb, Cr, and Mo without the presence of carbon (Table 3), suggesting the presence of intermetallic phases, similar to the case in Figure 2 and Table 2. Unfortunately, the quantitative analysis may have been altered by the XRD signal coming from elements contained in the matrix.

**Figure 6:**
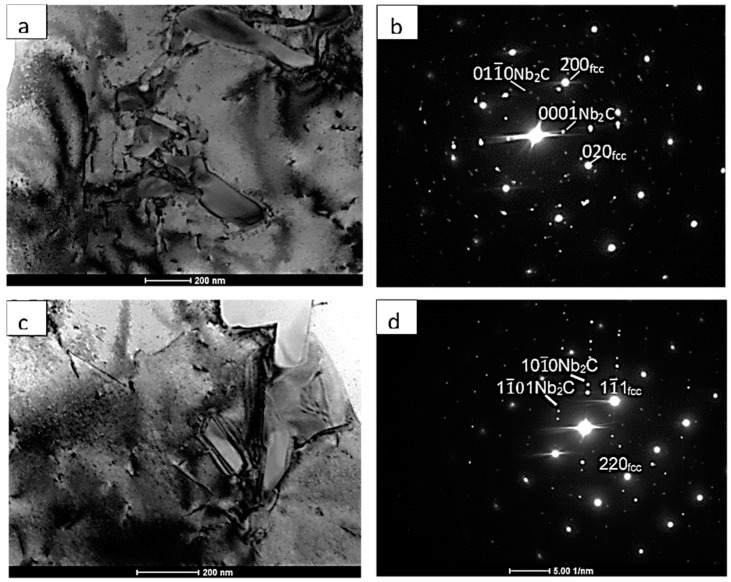
TEM micrographs (**a**,**c**) and corresponding SADP (**b**,**d**) for the deposited Inconel 625 using high-power CO_2_ laser.

To identify the structure of the precipitates and density of the dislocations, TEM studies were performed. Figure 6 shows TEM micrographs and the corresponding SADP from the melt pool region of the high-power CO_2_ laser-deposited Inconel 625 alloy. In agreement with the SEM observations, fine elongated particles of size 200–600 μm can be seen in Figure 6a,c. They show slightly darker or brighter contrast depending on orientation and well-defined boundaries due to noncoherent interfaces. Electron diffraction allowed the matrix to be identified as a γ-Ni disordered solid solution, while the particles could be identified as hexagonal Nb_2_C with a zone axis of [2$\bar{1}\bar{1}$0] in Figure 6b and [1$\bar{2}$1$\bar{3}$] in Figure 6d. Nb_2_C carbides have not yet been identified in additively manufactured Inconel 625 alloys. However, their shape, which is different from that of intermetallic phases observed in regularly manufactured Inconel 625, and the results of EDS microanalysis indicate that these carbides may form under certain cooling conditions. A low dislocation density characterizes the high-power CO_2_ laser deposition method, where low stresses were observed compared to those from the EBAM method, where an increased volume crystallization was also observed by Ma et al. [14]. It was shown that the residual stresses can be significantly minimized by reducing the layer thickness during additive manufacturing of various Inconel-type alloys.

**Figure 7:**
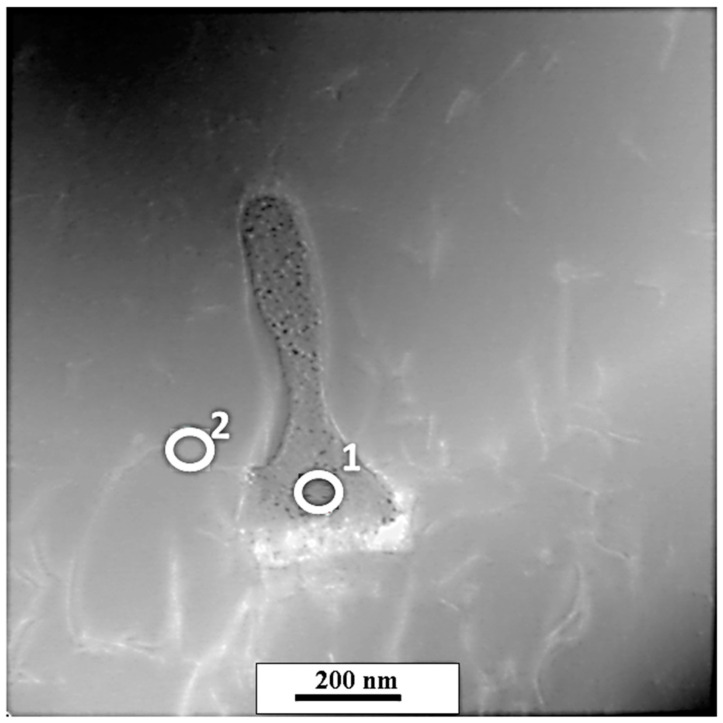
STEM micrograph of precipitation observed in the material deposited using high-power CO_2_ laser.

Figure 7 shows the results of STEM microanalysis of the precipitates and matrix from the CO_2_ laser-deposited sample. One can clearly see a slightly darker particle with an irregular shape. A dark contrast indicates the presence of heavy elements and, indeed, the EDS analysis shown in Table 4 indicates almost 67 at.% Nb in the precipitates compared to only 2 at.% in the matrix. This confirms the results of the SEM/EDS analysis and the results of the indexed TEM diffraction patterns, where Nb_2_C was identified. The small amounts of Mo, Ni, and Cr were in a solid solution and substituted for Nb in the carbides.

### 3.3. Microstructure of Inconel 625 Deposited Using LENS Technique

**Figure 8:**
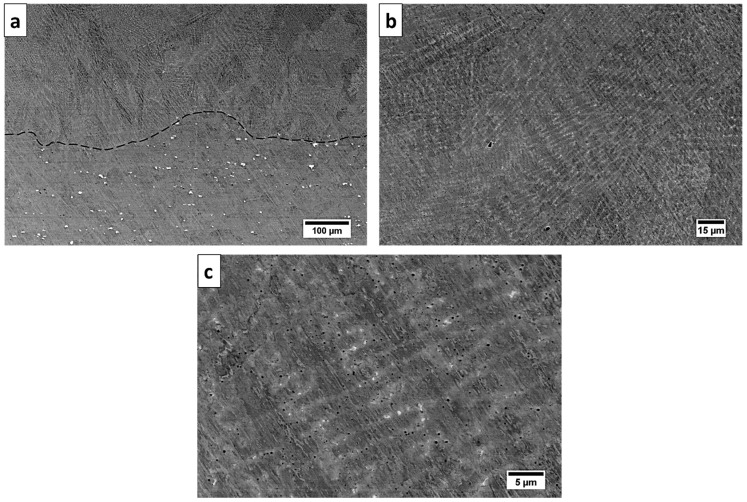
SEM micrograph of Inconel 625 deposited using laser-engineered net shaping (LENS) technique at three different magnifications: (**a**) low-magnification observations show the cross-section of a substrate and deposited material; (**b**) higher magnification of deposited material; (**c**) highest magnification showing the precipitations and porosity in the microstructure.

The microstructure of the Inconel 625 deposited using the LENS technique is presented in Figure 8a–c, which show SEM micrographs taken near the interface between the substrate and deposited material. An elongated cell-like structure with bright particles in the interdendritic areas can be seen. A bright contrast from the precipitates indicated that heavy elements gathered there.

**Figure 9:**
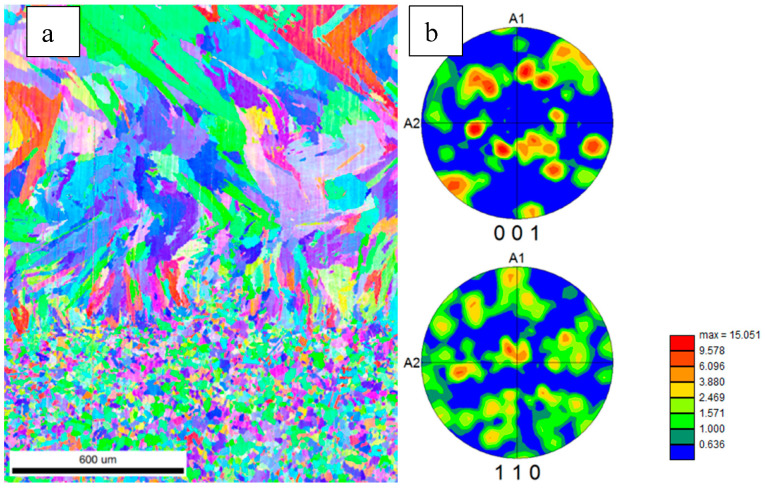
The EBSD results for samples fabricated using LENS technique: (**a**) inverse pole figure (IPF) maps for the substrate and the deposited material and (**b**) {001} and {111} pole figures rotated to represent the deposition surface; the color scale bar represents the intensity of the texture.

Figure 9 shows inverse pole figure (IPF) maps and calculated {100} and {110} pole figures from the deposited layer that were rotated to represent the deposition surface. One can see that the grains had a rather weak texture that was less developed than in the case for the high-power CO_2_ laser deposition method shown in Figure 4. Moreover, the grains were finer than in the case of the CO_2_ laser deposition method, and they were rotated by changing the layer cladding. This most likely occurred due to the lower power of the laser used in the experiment and smaller melt pool areas. This method also caused the formation of a different type of texture close to the {011} <100> Goss component, as observed in [14]. Moreover, from the grain boundary misorientation distribution angles, one can see that there were fewer HAGBs (66.9%) and more LAGBs (33.1%) than in the case of a higher-power laser due to the different heat flow conditions. The substrate also showed a larger number of low-angle grain boundaries (32.6%) since, at lower processing temperatures, they were not consumed by HAGBs (67.4%). The calculated average size of elongated grown crystallites was 95 μm, but the length of the crystals was smaller than in the case shown in Figure 4 and rotated from one to the other layer. A similar type of structure was observed in the case of Inconel 625 in [14], where the Goss component was observed.

**Figure 10:**
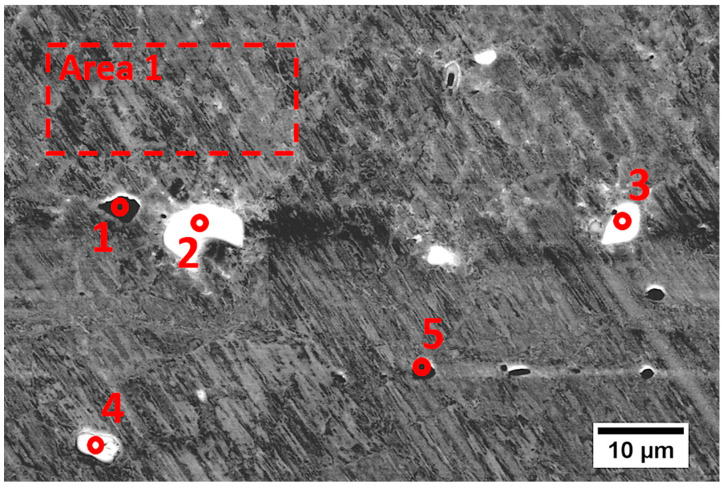
SEM (BSE) micrograph of precipitations observed in the material deposited using LENS technique.

Figure 10 shows an SEM micrograph with EDS chemical analysis (Table 5) from selected bright and dark particles and from the area of solid solution. Generally, in the interdendritic areas, particles rich in Nb, Mo, Cr, and Ti and poor in Ni were observed. However, additional carbon analysis from the bright and dark particles indicated rather high levels of carbon, which most likely indicated the presence of niobium or titanium carbides. The content of carbon was between 30 and 60 at.%; however, due to substantial contamination, a large measurement error occurred, and the carbon content could not be attributed to a particular carbide. Carbon was not detected in the Ni solid solution, and the results of the analysis were generally close to that of the alloy. The majority of the bright particles possessed a high amount of Nb and C with low contents of Ti and Cr, suggesting the presence of NbC, and the dark particles possessed more Ti and C with small amounts of Nb and Cr, suggesting the presence of TiC, in agreement with previous observations published in [2,4,7].

**Figure 11:**
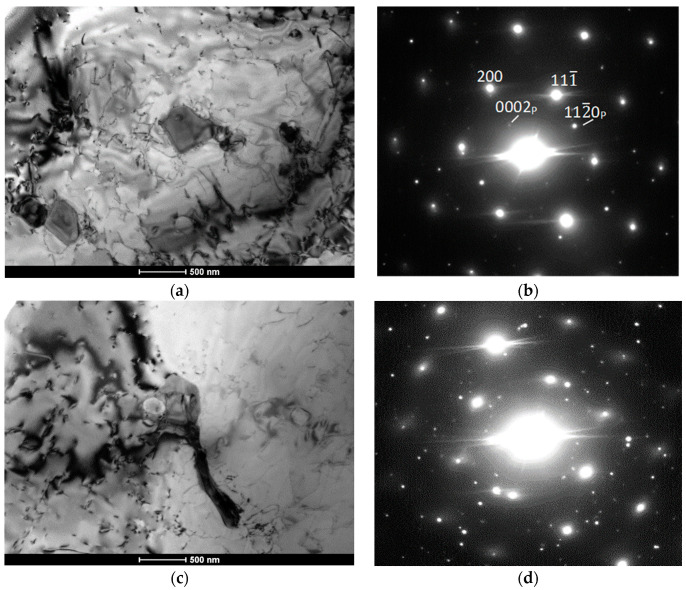
TEM micrograph and selected area diffraction pattern from the LENS deposited sample. (**a**,**b**) indexing of diffraction patter shows [011] zone axis of face-centered cubic (FCC) γ matrix and [1$\bar{1}$00] zone axis of the NiCrNb intermetallic hexagonal particle; (**c**,**d**) indexing of diffraction patter shows [011] zone axis of FCC matrix and [011] zone axis of the NbC cubic carbide particle.

To identify the crystal structure and chemical composition of the particles, TEM analytical and diffraction studies were performed. Figure 11 shows a TEM micrograph and selected area diffraction pattern from the LENS-deposited sample. Indexing of the diffraction pattern showed the [011] zone axis of the FCC matrix and the [1$\bar{1}$00] zone axis of the NiCrNb intermetallic hexagonal particle. Its identification as the intermetallic phase is in agreement with the results of EDS analysis, where particles containing a high percentage of Nb, Ni, and Cr without carbon were found, suggesting the presence of intermetallic phases. Indeed, the NiNbCr phase of the hexagonal structure is consistent with the diffraction pattern in Figure 11. This result, however, was different from that reported in previous papers [2,4,7,13,15,17], where MC, Nb_3_C, and Laves phases were observed, confirming the possibility of the formation of intermetallic phases, in addition to those identified earlier. A slightly increased density of dislocations near intermetallic particles resulted from stress formation due to differences in dimensional changes during cooling of the particle and the matrix.

**Figure 12:**
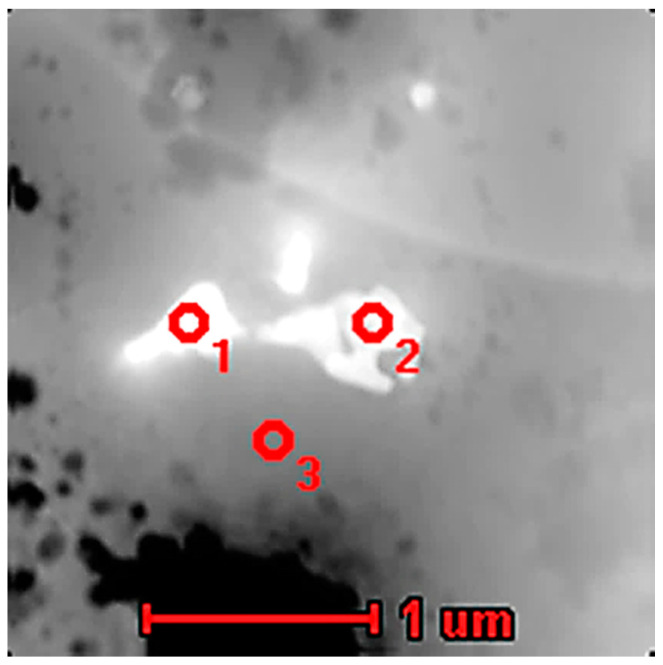
STEM micrograph of precipitation observed in the material deposited using LENS technique.

Figure 12 shows a STEM micrograph where two different particles with a size near 0.5 μm can be seen. The results of the EDS analysis shown in Table 6 suggested the presence of two types of particles. The first one had a rather rectangular shape and a high a Nb content with Mo and Cr and no carbon, which suggested an intermetallic phase. The second particle with a round shape contained some carbon and a high percentage of Nb, Cr, Ni, and Mo. These results suggest the presence of either an intermetallic or a carbide phase.

**Figure 13:**
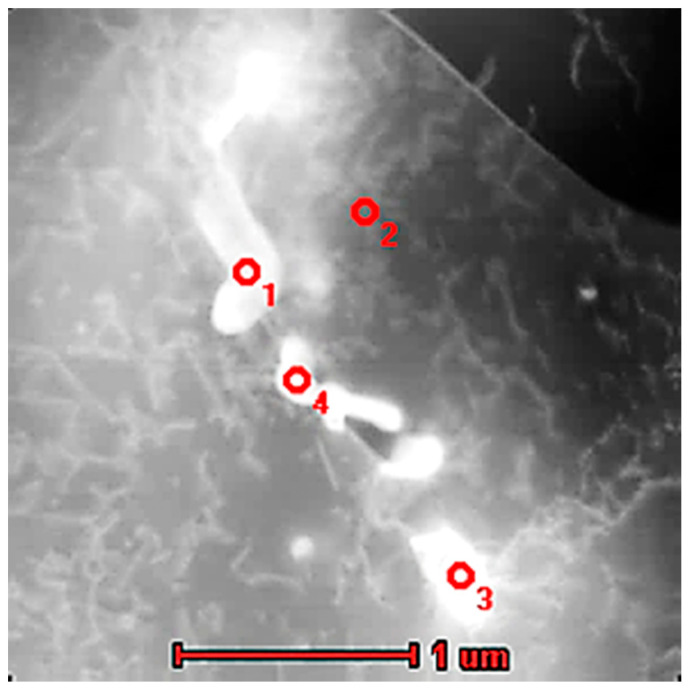
STEM micrograph of precipitation observed in the material deposited using LENS technique.

The next STEM micrograph (Figure 13) also shows various types of particles of rectangular or rounded shapes. The rounded irregularly shaped particles contained more carbon (Table 7), suggesting that they may be carbides, while those with orthogonal shapes did not contain carbon and indicated the likely presence of intermetallic phases. A higher than expected content of Ni may have resulted from the signal from the matrix in the case of small particles. Next, the TEM micrograph showed elongated particles, giving several diffraction patterns from a particle. One of them could be clearly identified as NbC with an [011] zone axis orientation, as also observed in previous works on AM Inconel 625 alloy [7,15,17]. 

### 3.4. Mechanical Properties of Inconel 625 Deposited Using High-Power CO_2_ Laser and LENS Technique

**Figure 14:**
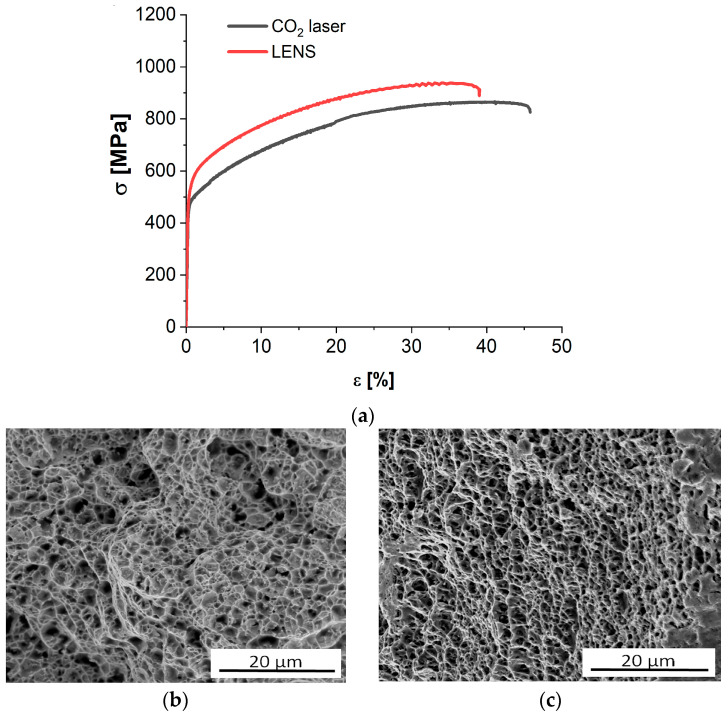
Tensile curves (**a**) and fracture surface (**b**,**c**) for Inconel 625 manufactured using high-power CO_2_ laser and LENS technique, respectively.

Figure 14a shows tensile curves for Inconel 625 prepared using a high-power CO_2_ laser and the LENS method tested at room temperature (RT). Both variants had the same slope with a proportional length, but the LENS-deposited Inconel 625 had a higher yield strength (YS; ~10%) and ultimate tensile strength (UTS; ~8%) and approximately 20% lower elongation than the samples fabricated using a high-power CO_2_ laser (Table 8). This was the effect of the cooling rate, which reached 1.7 × 10^4^ s^−1^ for the LENS process and was over 10 times higher than that for the high-power CO_2_ laser method. The influence of the cooling rate on the Inconel 625 mechanical properties was also proven by the microhardness measurements. It was noted that the average LENS-deposited Inconel 625 sample hardness was 55 HV0.1 higher than that of the CO_2_ laser variant (Table 8). 

A representative fracture surface after tensile tests within different regions is shown in Figure 13. The fractography of all samples was dominated by a transgranular fracture, which is a typical feature of ductile failure. The fracture surface comprised typical dimples of different sizes spread throughout. The higher mechanical properties of LENS method-prepared samples resulted most probably from stronger solid solution hardening manifested by increased content of alloying elements in the sample due to a higher cooling rate when using a lower-power LENS laser. The size of carbide and intermetallic particles was similar in samples prepared using both methods, and their role in hardening of samples was estimated as insignificant. 

## 4. Conclusions

1. A comparison of two types of direct deposition systems, one with a high-power CO_2_ laser (2200 W) that transported the powder through helium gas to the laser beam focal point and injected it into the melt pool and the other with a LENS system of a lower power (400 W) and processed in the chamber under a purified argon atmosphere, was performed in view of differences in resulting microstructure and mechanical properties. The high-power laser application resulted in a strong <100> build texture, while, at a low power, the Goss component {011} <100> increased. This orientation was accompanied by an increased number of low-angle grain boundaries.
2. Due to the higher cooling rate of the melt pool during the LENS process, the obtained samples showed higher yield strengths, ultimate tensile strengths, and a lower plasticity than the samples fabricated by the high-power CO_2_ laser deposition due to a higher supersaturation of alloying elements and solid solution strengthening mechanism in addition to precipitate hardening. Moreover, a higher average microhardness (by approximately 55 HV0.1) for the LENS-deposited Inconel 625 samples was observed.
3. Both types of deposited materials had dendritic microstructures with Ti-, Mo-, and Nb-rich zones at cell boundaries, where numerous precipitates were also observed. The elongated grains in the build directions were larger than those in the substrate and had a length that reached 1 mm. The average size was 95 μm compared to 31 μm for the substrate. The low-power laser caused similar sizes for the deposited grains elongated in the <100> growth direction, but they were inclined with respect to the substrate by 45°.
4. The main precipitates identified using SEM EDS and STEM microanalysis were Nb_2_C and NbC carbides with a high percentage of Mo, Cr, and Ti. The microanalysis results also suggested the presence of titanium carbides. In addition, the results of the EDS analysis suggested the presence of intermetallic compounds, such as Nb_3_Ni and NbNiCr. The latter was identified using electron diffraction.

## Figures and Tables

**Table 1:** Chemical composition of powder feedstock used in both laser metal deposition systems.

Chemical Composition (at.%)										
Ni	Cr	Nb	Mo	Si	Mn	Fe	Co	Al	Ti	C
58.0	21.6	3.6	1.7	0.5	0.5	4.9	1.0	0.4	0.4	0.1

**Table 2:** Chemical composition of selected precipitations and the marked area presented in Figure 2.

No	Chemical Composition (at.%)								
	Ni	Cr	Nb	Mo	Si	Fe	Al	Ti	C
1	3.1	1.3	52.2	1.7	0.6	–	0.2	3.8	37.0
2	4.4	2.1	49.2	1.4	0.6	–	0.2	4.9	37.3
3	2.1	3.2	19.9	0.3	–	–	–	74.5	–
Area	63.0	25.0	1.7	4.1	0.3	5.2	0.3	0.4	–

**Table 3:** Chemical composition of selected precipitations and the marked area presented in Figure 5.

No	Chemical Composition (at.%)								
	Ni	Cr	Nb	Mo	Si	Fe	Al	Ti	C
1	41.8	21.7	13.9	8.8	4.0	0.8	0.1	0.2	8.7
2	35.5	21.1	19.0	8.4	3.7	0.4	0.2	0.3	11.4
3	45.1	24.0	15.4	10.1	4.5	0.6	0.3	–	–
Area 1	62.8	22.6	1.1	3.5	0.6	1.1	0.2	–	7.6
Area 2	63.2	23.2	1.3	3.7	0.7	1.1	0.1	0.1	6.6

**Table 4:** Chemical composition of selected precipitations and the marked area presented in Figure 7.

No	Chemical Composition (at.%)				
	Ni	Cr	Nb	Mo	C
1	1.8	4.7	66.8	2.3	24.3
2	56.0	15.7	2.0	5.3	20.9

**Table 5:** Chemical composition of selected precipitations and the marked area presented in Figure 10.

No	Chemical Composition (at.%)						
	Ni	Cr	Nb	Mo	Fe	Ti	C
1	1.4	2.0	6.7	–	0.5	47.9	40.5
2	1.2	0.7	30.6	–	0.3	3.4	60.8
4	8.5	4.4	32.3	–	0.9	4.8	48.9
5	2.6	3.1	7.1	0.5	0.8	50.0	35.5
Area	61.8	26.0	1.7	4.0	4.1	0.3	–

**Table 6:** Chemical composition of selected precipitations and the marked area presented in Figure 12.

No	Chemical Composition (at.%)					
	Ni	Cr	Nb	Mo	Fe	C
1	3.3	11.4	79.7	5.5	–	–
2	34.7	15.6	26.3	18.7	2.6	1.7
3	10.8	20.5	2.3	6.4	2.7	18.0

**Table 7:** Chemical composition of selected precipitations and the marked area presented in Figure 13.

No	Chemical Composition (at.%)					
	Ni	Cr	Nb	Mo	Fe	C
1	39.5	17.6	79.7	13.9	–	11.9
2	58.3	24.4	3.0	7.4	6.9	–
3	55.4	–	36.5	6.7	–	1.4
4	47.6	21.2	11.0	9.7	3.9	6.4

**Table 8:** Mechanical properties and cooling rate of samples fabricated by using high power CO_2_ laser and LENS methods.

Fabrication Method	YS (MPa)	UTS (MPa)	Elongation (%)	Microhardness (HV0.1)	Cooling Rate (s^−1^)
CO_2_ laser	472 ± 1	868 ± 3	42 ± 4	261 ± 6	1.5 × 10^3^
LENS	524 ± 13	944 ± 12	35 ± 4	316 ± 5	1.7 × 10^4^
